# Insulin allergy can be successfully managed by a systematic approach

**DOI:** 10.1186/s13601-018-0223-x

**Published:** 2018-09-25

**Authors:** Maija Bruun Haastrup, Jan Erik Henriksen, Charlotte Gotthard Mortz, Carsten Bindslev-Jensen

**Affiliations:** 1 Department of Clinical Biochemistry and Pharmacology, Odense University Hospital, Odense, Denmark; 2 Steno Diabetes Center Odense, Odense University Hospital, Odense, Denmark; 3 Odense Research Center for Anaphylaxis (ORCA), Department of Dermatology and Allergy Centre, Odense University Hospital, Odense, Denmark

**Keywords:** Insulin, Allergy, IgE, Intracutaneous test

## Abstract

**Background:**

Type I insulin allergy can be a challenging condition, and there is no international consensus on how to establish the diagnosis. Measurement of specific IgE and skin testing have been cornerstones in the diagnostic work-up. However, these tests have limitations, mainly lack of correlation between test results and clinical findings. At the Allergy Centre, Odense University Hospital, patients with suspected insulin allergy have been evaluated since 2003. The aim of this study was to establish a systematic approach to diagnose and treat patients with insulin allergy.

**Methods:**

The study was conducted retrospectively by retrieving data from the Allergy Centre database on patients with suspected insulin allergy evaluated from 2003 to 2017. The examination comprised a comprehensive medical history, specific IgE against insulin and intracutaneous tests (ICT) with different insulins.

**Results:**

A total of 144 patients were examined on suspicion of insulin allergy of which 110 had negative specific IgE in serum. Of the remaining 34 patients, 33 had ICT performed; 2 had negative ICTs, while 31 had one or more positive ICT. All 34 patients had mild symptoms, and 4 could obtain symptom relief with antihistamines or local steroids, 9 could be managed with oral antidiabetics, and 7 were switched to other insulins. The final 14 patients were offered an insulin pump because of reactions to many different insulins, many positive ICTs, unmanageable diabetes, young age and compliance, or convenience.

**Conclusion:**

Insulin allergy can be managed by a systematic approach, and symptom relief is obtainable in most patients.

## Background

Insulin allergy affects 0.1–3% of insulin-treated diabetics [1, 2] and causes symptoms ranging from localized itching and rash to life-threatening anaphylaxis [3–5]. The IgE-mediated (type I) reaction is by far the most common, but type III and type IV reactions have been reported as well [1, 6–9].

The diagnosis is based on past and present symptoms and signs, together with skin tests and specific immunoglobulin E (IgE) measurement in serum. Skin prick test (SPT) and intracutaneous test (ICT) have traditionally been used in the evaluation of these patients. However, the reliability of the results from skin tests has been questionable for a number of reasons including false-negative tests, and non-specific reactions as well as reactions to additives (e.g. protamine sulfate) [3, 5, 6, 8–10]. Measurement of specific IgE (sIgE) is another cornerstone in the diagnosis, but this method has limitations as well, mainly due to poor correlation between clinical findings and elevated IgE levels [3, 6]. Consequently, there is no consensus on the correct method for diagnosing insulin allergy yet, though one was suggested by Jacquier et al. in 2013 [11], based on three patient cases. The authors suggested the use of measurement of total IgE, insulin-specific IgE, and anti-insulin antibodies (IgG) in addition to SPT or ICT.

The treatment of insulin allergy is often straightforward. For many patients it is possible to switch insulin preparation or to avoid insulin use by managing their diabetes through diet or oral antidiabetics and/or injections with glucagon-like peptide 1 (GLP1) analogue treatment. Some patients, however, are insulin dependent and experience symptoms during treatment with many different insulins. These patients are difficult to treat and require a more comprehensive approach, sometimes including desensitization [3, 9, 12].

At the Allergy Centre at Odense University Hospital patients with insulin allergy have been diagnosed and treated since 2003. The aim of the present study was to summarize the diagnostic findings and to present a systematic approach for the examination and treatment of these patients.

## Methods

This study was conducted retrospectively and included data from the Allergy Centre database, ACbase, on patients seen at the Odense Research Center for Anaphylaxis (ORCA), Allergy Centre at Odense University Hospital from 2003 through March 2017 with suspicion of insulin allergy. Since 2010 the Allergy Centre in Odense has been the only place in Denmark to evaluate patients suspected of insulin allergy in collaboration with the Department of Endocrinology, Odense University Hospital.

The examination comprised a comprehensive medical history (type of diabetes, duration and severity of symptoms, prior treatment etc.), measurement of specific IgE against human, bovine, and porcine insulin and—where appropriate—also ICT with different insulins.

Specific IgE measurement (ImmunoCAP) against human, bovine and porcine insulin was performed by Thermo Fisher, Uppsala Sweden. Values above 0.35 kIU/L were considered positive.

Intracutaneous tests were performed by injecting 20–50 µL of the different insulins in the concentration 5 IE/mL, and reactions were considered positive if the wheal size diameter was 3 mm larger than the initial bleb. ICTs were read after 20 min, according to guidelines from the European Network on Drug Allergy (ENDA) [13]. As controls, skin prick tests were performed with histamine 10 mg/mL (ALK-Abello, Denmark) as the positive control and isotonic NaCl as the negative control.

The insulins available for the test varied over time, and some of the first patients seen (n = 3) were only tested with a selection of the available insulin types, based on the clinician’s judgment. The majority, however, were tested with the full panel of insulins, which included rapid acting human insulin (Humulin Regular^®^, Insuman Rapid^®^, Actrapid^®^), rapid acting analogue insulin lispro (Humalog^®^), and insulin aspart (NovoRapid^®^), and intermediate acting isophane human insulin (Humulin NPH^®^, Insulatard^®^, both containing protamine), and long acting analogue insulin: Insulin glargine (Lantus^®^), and insulin detemir (Levemir^®^). Most recently, the following additions have been made to the panel: Toujeo^®^, Abasaglar^®^ (both insulin glargine), Tresiba (insulin deglucec), NovoMix^®^ (insulin aspart with added protamine), and Apidra^®^ (insulin glulisine).

SPT with insulin was initially a part of the examination but was abandoned due to the poor sensitivity and specificity of the test. Basophil histamine release (HR test) was performed in a few patients but not systematically. Consequently, the results of SPT and HR test are omitted.

The patients—or in the case of children, their parents—gave informed consent to store their data in the ACbase. All data for this study were collected after approval from the Danish Data Protection Agency (17/11270).

## Results

**Fig. 1:**
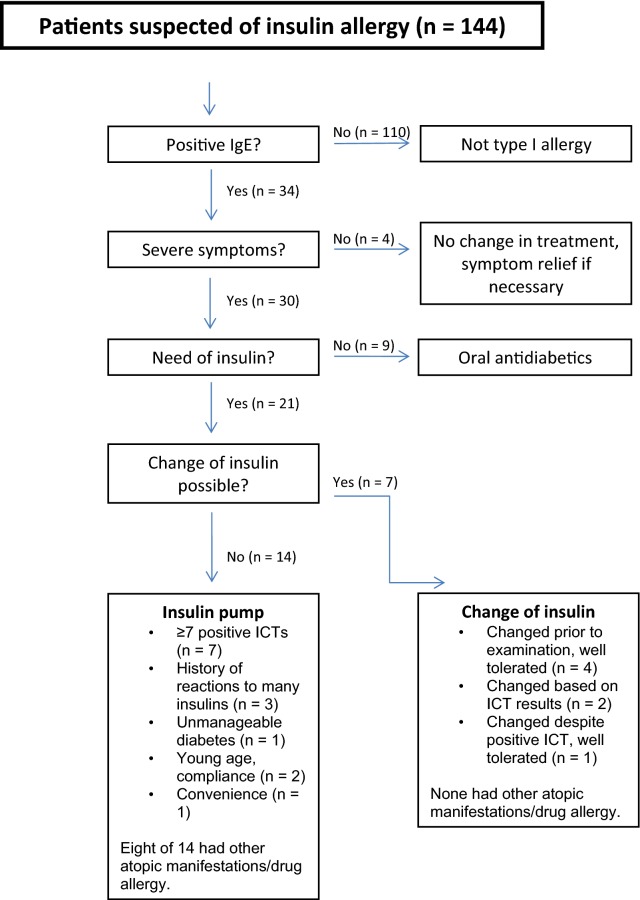
Flow chart of the procotol for examination of suspected insulin allergy

A flow chart of the protocol for the examination of suspected insulin allergy is presented in Fig. 1.

A total of 144 patients with suspected insulin allergy were seen at the Allergy Centre, Odense University Hospital, from 2003 through March 2017. Of these, 34 had positive sIgE for insulin and were included in the study while 110 had negative sIgE for insulin and were judged non-allergic (type I). Among those deemed non-allergic 71 had an ICT performed with 12 having one or more positive ICTs. Another 5 were positive to protamine sulfate but none of the included insulins. ICTs were performed in these patients before the results of the IgE measurements were available. A positive ICT in a patient with a negative IgE may be an unspecific reaction, a reaction to an additive, or a delayed reaction reflecting a type IV allergy.

The 34 patients with positive sIgE were categorised according to symptom severity. All 34 patients had local symptoms only (pruritus, nodules, localised dermatitis and infiltration at injection sites). Patients, whose symptoms could be alleviated by antihistamines or local steroids (n = 4), and patients, who could be managed by other antidiabetic medications than insulin (n = 9), were treated accordingly.

In 33 of the 34 patients with positive sIgE, ICT was performed. One was not tested due to age (12 years). Two of the 33 patients exhibited negative ICTs, however one of the two was only tested for one specific insulin preparation (Apidra^®^) due to age (9 years).

The demographics and test results of the 21 insulin dependent patients with positive sIgE are presented in Table 1 (data not shown for the remaining 13 patients because the symptoms of these patients could be managed without insulin). Of the 20 patients tested with ICTs 19 were ICT-positive for at least one insulin preparation (range 1–9). The ICT-negative patient was the 9-year old girl, who was only tested for one insulin preparation by ICT.

**Table 1:** Demographics and test results

ID	Sex	Age	DM type	sIgE			ICT									
				Porcine	Bovine	Human	A	B	C	D	E	F	G	H	I	J
1	F	60	II	2.1	1.3	1.6	–	+	–	–	–	–	–	–	–	N/D
2	F	55	II	0.6	0.4	0.5	+	N/A	N/A	N/A	N/A	N/A	N/A	+	+	N/D
3	F	14	I	2.6	2.0	1.9	+	+	–	–	+	+	N/D	+	+	N/D
4	F	59	II	7.1	5.9	6.2	+	+	+	+	+	+	+	N/D	N/D	N/D
5	F	41	II	1.5	1.1	1.8	+	+	+	+	+	+	+	+	+	N/D
6	M	55	II	22.8	18.5	22.8	+	+	+	+	+	+	+	+	+	N/D
7	M	67	II	0.6	0.4	0.5	+	+	+	+	+	+	+	+	–	N/D
8	F	47	II	8.9	7.9	7.7	+	+	+	+	+	+	+	N/D	–	N/D
9	M	37	II	0.4	< 0.35	0.4	–	+	–	–	–	–	–	–	+	N/D
10	F	36	I	1.3	1.0	1.0	–	–	–	–	–	–	+	–	–	N/D
11	F	37	II	1.7	1.3	1.6	+	+	+	+	+	+	+	+	+	N/D
12	F	12	I	0.4	< 0.35	< 0.35	N/D	N/D	N/D	N/D	N/D	N/D	N/D	N/D	N/D	N/D
13	F	9	I	0.5	< 0.35	0.4	N/D	N/D	N/D	N/D	N/D	N/D	N/D	N/D	N/D	–
14	F	54	I	0.1	< 0.35	0.4	–	–	N/D	–	–	–	–	+	–	N/D
15	F	64	I	2.9	2.4	2.7	+	+	+	N/A	N/A	N/A	N/A	+	+	N/D
16	F	46	II	2.5	2.2	2.2	+	+	+	N/D	N/D	N/D	N/D	+	N/D	N/D
17	M	56	II	2.4	2.1	1.8	+	+	+	+	+	+	+	+	+	N/D
18	M	55	I	0.4	< 0.35	0.4	–	–	–	–	–	–	+	–	–	N/D
19	M	30	I	3.4	2.5	2.2	+	+	+	–	–	+	+	–	+	N/D
20	M	57	I	0.6	0.6	0.7	+	–	+	–	+	–	–	–	–	N/D
21	M	79	II	15.0	15.2	13.2	N/D	+	–	+	+	+	+	+	+	N/D

Patients 1–14 were given an insulin pump, 15–21 changed insulin preparation. All symptoms were local. *N/A* result not available in the database, *N/D* not done. IgE > 0.35 was considered positive

*A* humulin regular, *B* insuman rapid, *C* actrapid, *D* humulin NPH, *E* insulatard, *F* lantus, *G* levemir, *H* novorapid, *I* humalog, *J* apidra

In the 21 insulin dependent patients a change in treatment regimen was necessary due to symptom severity. Where possible (n = 7), a switch was made to another insulin preparation. Of these 7 patients, a well-tolerated switch had already been made prior to evaluation at the Allergy Centre in 4, and consequently, no further treatment adjustment was needed. Two were successfully switched to a different insulin, based on negative ICT. The last patient in this group was switched to an insulin despite a positive ICT for this particular insulin. The switch, however, was well-tolerated.

The final group of patients (n = 14) was offered treatment with an insulin pump in collaboration with the Department of Endocrinology, Odense University Hospital. Seven of these patients exhibited ≥ 7 positive ICTs. Three additional patients had histories of reactions to many different insulins. One patient’s diabetes was unmanageable, two were offered an insulin pump because of young age and compliance and one due to convenience.

Of the 14 patients given an insulin pump, eight had other atopic manifestations or drug allergy (challenge-verified penicillin allergy, atopic dermatitis, asthma, hay fever, contact allergy, and urticaria). None of the 7 patients, whose insulins were changed, had other atopic manifestations/other drug allergies. The groups did not differ with regards to age, sex, diabetes type, sIgE levels or number of positive ICTs, see Table 2.

**Table 2:** Comparison of patient characteristics and test results between the two groups

	Insulin pump (n = 14)	Other insulin (n = 7)
Sex (M:F)	3:11	5:2
Age (years)	41.6 (9–67)	55.3 (30–79)
Diabetes type (I:II)	4:10	4:3
Other atopic manifestations/drug allergy (Y:N)	8:6	0:7
No. of positive ICTs	5.9 (0–9)	5 (1–9)
IgE_human_ (kIU/L)	4.07 (0.4–22.8)	3.31 (0.4–13.2)
IgE_bovine_ (kIU/L)	4.76 (0.4–18.5)	4.17 (0.6–15.2)
IgE_porcine_ (kIU/L)	3.64 (0.4–22.8)	3.89 (0.4–15.0)

Overview of sex distribution and type I/type II diabetes ratio, average age, sIgE (human, bovine and porcine) and number of positive ICTs (range). Other atopic manifestations/drug allergy: Two patients had challenge-proven penicillin allergy, 2 asthma, 2 hay fever, 1 urticaria and 1 atopic dermatitis and contact allergy. Only positive sIgE values are included

## Discussion

The majority of the published literature concerning insulin allergy has been case reports or small case series. This study is the largest of its kind so far.

Of a total of 144 patients suspected of insulin allergy only 34 (24%) had positive sIgE, which is comparable to a study by Bodtger and Wittrup from 2005 [14], where the diagnosis was established in 9 of 22 patients (41%). The diagnostic work-up in this study consisted, however, only of intracutaneous testing.

We focused mainly on specific IgE measurement for the diagnosis of insulin allergy, because this in vitro test has a high negative predictive value [10, 15], whereas elevated levels are not necessarily indicative of allergy and are thus of limited value without a thorough medical history and supplementary skin testing [9, 16].

Skin tests, particularly SPT, have poor sensitivity but high specificity, and the negative predictive value of ICT is high [5, 9, 15]. These tests can be helpful in distinguishing between insulins with and without the capability of causing clinical reactions when choosing future treatment for a given patient.

A total of 13 of the 34 patients (38%) with an insulin allergy diagnosis in our study could be managed without a change in treatment or with oral antidiabetics. Another 7 were switched to a different insulin preparation, and the final 14 were desensitised with continuous subcutaneous infusions through an insulin pump. This has been described in several reports as an option for patients with an indispensable need for insulin who cannot be managed by switching to another insulin preparation [8, 17–22].

The most recent addition to the treatment options for these patients is omalizumab. This has been successful in two of three case reports so far and may represent an interesting alternative for patients whose symptoms cannot otherwise be managed [23–25].

None of the patients in the present study had a history of systemic reactions to insulin. Therefore, other treatment options than desensitization were chosen whenever possible. An insulin desensitization was mainly performed in those with many positive ICTs and a history of reactions to many different insulin preparations. In case of a history of anaphylaxis in insulin dependent patients, desensitization should always be performed.

Type 2 diabetics potentially represent a twofold challenge given that their endogenous insulin production decreases with time which most likely will cause a return of the symptoms of insulin allergy, when the patients can no longer be managed with oral antidiabetics.

## Conclusion

Insulin allergy is a challenging condition, but can most often be managed by a systematic approach, and symptom relief is obtainable in most if not all patients.

## Abbreviations

ENDA: European Network on Drug Allergy
ICT: intracutaneous test
IgE: immunoglobulin E
ORCA: Odense Research Center for Anaphylaxis
sIgE: specific IgE
SPT: skin prick test
